# Cerebral perfusion variance in new daily persistent headache and chronic migraine: an arterial spin-labeled MR imaging study

**DOI:** 10.1186/s10194-022-01532-7

**Published:** 2022-12-08

**Authors:** Xiaoyan Bai, Wei Wang, Xueyan Zhang, Zhangxuan Hu, Yingkui Zhang, Zhiye Li, Xue Zhang, Ziyu Yuan, Hefei Tang, Yaqing Zhang, Xueying Yu, Peng Zhang, Yonggang Wang, Binbin Sui

**Affiliations:** 1 Tiantan Neuroimaging Center for Excellence, China National Clinical Research Center for Neurological Diseases, Beijing Tiantan Hospital, Capital Medical University, No.119 South Fourth Ring West Road, Fengtai District, Beijing, 100070 China; 2 Department of Radiology, Beijing Tiantan Hospital, Capital Medical University, No.119 South Fourth Ring West Road, Fengtai District, Beijing, 100070 China; 3 Headache Center, Department of Neurology, Beijing Tiantan Hospital, Capital Medical University, No.119 South Fourth Ring West Road, Fengtai District, Beijing, 100070 China; 4 Department of Neurology, The First Affiliated Hospital of Zhengzhou University, No.1 Jianshe East Road, Zhengzhou, Henan Province, 450000 China; 5 GE Healthcare, No.1 Tongji Nan Road, Beijing Economic Technological Development Area, Beijing, 100176 China

**Keywords:** New daily persistent headache, Chronic migraine, Perfusion, Magnetic resonance imaging, Arterial spin labeling

## Abstract

**Background and purpose:**

New daily persistent headache (NDPH) and chronic migraine (CM) are two different types of headaches that might involve vascular dysregulation. There is still a lack of clarity about altered brain perfusion in NDPH and CM. This study aimed to investigate the cerebral perfusion variances of NDPH and CM using multi-delay pseudo-continuous arterial spin-labeled magnetic resonance imaging (pCASL-MRI).

**Methods:**

Fifteen patients with NDPH, 18 patients with CM, and 15 age- and sex-matched healthy controls (HCs) were included. All participants underwent 3D multi-delay pCASL-MRI to obtain cerebral perfusion data, including arrival-time-corrected cerebral blood flow (CBF) and arterial cerebral blood volume (aCBV). The automated anatomical labeling atlas 3 (AAL3) was used to parcellate 170 brain regions. The CBF and aCBV values in each brain region were compared among the three groups. Correlation analyses between cerebral perfusion parameters and clinical variables were performed.

**Results:**

Compared with HC participants, patients with NDPH were found to have decreased CBF and aCBV values in multiple regions in the right hemisphere, including the right posterior orbital gyrus (OFCpost.R), right middle occipital gyrus (MOG.R), and ventral anterior nucleus of right thalamus (tVA.R), while patients with CM showed increased CBF and aCBV values presenting in the ventral lateral nucleus of left thalamus (tVL.L) and right thalamus (tVL.R) compared with HCs (all *p* < 0.05). In patients with NDPH, after age and sex adjustment, the increased aCBV values of IFGorb. R were positively correlated with GAD-7 scores; and the increased CBF and aCBV values of tVA.R were positively correlated with disease duration.

**Conclusion:**

The multi-delay pCASL technique can detect cerebral perfusion variation in patients with NDPH and CM. The cerebral perfusion changes may suggest different variations between NDPH and CM, which might provide hemodynamic evidence of these two types of primary headaches.

**Supplementary Information:**

The online version contains supplementary material available at 10.1186/s10194-022-01532-7.

## Introduction

New daily persistent headache (NDPH) is a new-onset primary headache with a clearly remembered onset, characterized by persistent headache that is continuous and unremitting within 24 hours and then persists on a daily basis for more than 3 months [1]. In community-based settings, two studies from Spain and Norway reported that the 1-year prevalence of NDPH was 0.1% [2] and 0.03% [3], respectively. Although rare, NDPH is one of the most treatment-refractory primary headache disorders, and may significantly affect the individual’s quality of life and can lead to psychiatric comorbidity [4]. Similarly, as a disabling disease, Chronic migraine (CM) is defined as headaches on at least 15 days per month for more than 3 months, with at least eight headache days per month fulfilling the criteria for migraine headaches [1]. It affects 1.4–2.2% of the general population and is a significant impact on the socioeconomic functioning and life quality of patients [5]. These two types of headache diseases have drawn more and more attention in late years, but the underlying pathophysiology mechanisms of NDPH and CM are still unclear. The elucidation of the underlying pathophysiological mechanisms will help early accurate diagnosis and treatment strategies.

The pathophysiology mechanisms of migraine chronification, including the atypical pain processing, cortical hyperexcitability, inflammation and central sensitization, have been studied [6, 7]. And the pathogenesis of NDPH was thought to be related to the stimulation of inflammatory factors secondary to CNS inflammation [2]. However, in recent years, more and more ground has been received by the “neurovascular hypothesis”, assuming an interplay of both vascular and neuronal factors to be involved in the development of migraine [8, 9]. Some studies have shown that headache is a neurovascular disease, and abnormal hemodynamics caused by neurovascular dysfunction may be one of the pathological mechanisms of headache [9–12]. A recent CM study using 3D pseudo-continuous arterial spin labeling (3D pCASL) imaging detected hypoperfusion of the left nucleus accumbens [10]. However, cerebral hemodynamic investigation was still rarely reported in CM, and there was no report of the cerebral hemodynamic features in NDPH. As we all know, NDPH and CM are both primary chronic headache disorders, but some evidence suggested that NDPH has distinct clinical features, risk factors, therapeutic options, and prognosis compared with CM [2, 6]. At present, the comparative study of the hemodynamic status in these two types of primary chronic headaches has not been reported. Therefore, we would like to investigate whether these two types of chronic headaches would present with hemodynamic variances. In particular, we would like to focus on the hemodynamic status of NDPH and compare whether there are different hemodynamic characteristics between NDPH and CM in this preliminary study.

Arterial spin labeling (ASL) is a magnetic resonance (MR) imaging technique that enables assessing brain perfusion without applying an exogenous contrast agent. The reliability and reproducibility of ASL in cerebral perfusion measurement have been validated, with ASL results showing consistency with positron emission tomography (PET) and dynamic susceptibility contrast (DSC) [13–15]. Three-dimensional pseudo-continuous ASL (pCASL) has been adopted as the evaluation method of cerebral hemodynamics in clinical practice due to its ease of implementation and high signal-to-noise ratio (SNR) [16]. It has been widely applied in dementia, stroke, vascular malformations, and tumors [16–20]. Several studies have reported on the cerebral perfusion changes in episodic migraine and found abnormal regional hyperperfusion in gray matter [12, 21, 22]. Multi-delay pCASL, in which images with several post-label delay (PLD) times are taken for improvement of the accuracy of cerebral blood flow (CBF) quantification, has been applied to ischemic stroke, moyamoya disease, and idiopathic generalized epilepsy [23–25]. However, so far, less is known about the regional cerebral perfusion in primary chronic headache based on multi-delay 3D pCASL MR imaging (pCASL-MRI). We hypothesized that abnormal regional cerebral perfusion presents in NDPH and CM compared with HC, and cerebral perfusion characteristics differ between NDPH and CM.

In this study, we aimed to investigate the cerebral perfusion variance of NDPH and CM using multi-delay pCASL-MRI, and to evaluate the relationship between the cerebral perfusion parameters and clinical variables.

## Methods

### Participants

From October 2020 to April 2022, 16 healthy control participants (HC) and 35 patients who were diagnosed NDPH (*n* = 15) and CM (*n* = 20) were enrolled in the Headache Center, Department of Neurology, Beijing Tiantan Hospital, Capital Medical University. This work was approved by the Institutional Review Board (KY2022–044) of Beijing Tiantan Hospital, Capital Medical University. All participants provided written informed consent prior to study enrolment. The study was registered with https://www.clinicaltrials.gov (unique identifier: NCT05334927). All of patients with CM were migraine without aura. The inclusion criteria for NDPH and CM were (1) the patients satisfy the definition of NDPH and CM according to the 3rd edition of the International Classification of Headache Disorders (ICHD-3) [1], (2) all participants ranged in age from 20 to 70 years. The exclusion criteria of NDPH and CM were as follows: (1) with other types of primary headache, (2) MRI claustrophobia or contraindications, (3) poor image quality, (4) history of alcohol or substance abuse, (5) brain damage or other neurological diseases (such as epilepsy, stroke, and physical disease) that can affect research results. The inclusion criteria for HCs were: (1) feasibility of MRI scan (no claustrophobic syndrome and no metal in the body); (2) no neurological or other major systemic diseases; (3) match age and sex to patients of NDPH and CM. The exclusion criteria of HCs were as follows: (1) pregnancy or breastfeeding; (2) MRI contraindications; (3) poor MRI data quality.

Demographic data were recorded for all participants. Clinical scales including the Headache Impact Test-6 (HIT-6), Patient Health Questionnaire-9 (PHQ-9), Generalized Anxiety Disorder-7 (GAD-7), and Pittsburgh Sleep Quality Index (PSQI) were assessed by an experienced neurologist and recorded in our headache questionnaire before the MRI data acquisition. HIT-6 [26] is designed to measure the impact and effect of headache on the ability to function normally in daily life, PHQ-9 [27] is designed to measure symptoms of depression in primary care settings, GAD-7 [28] is used to assess anxiety, and PSQI [29] is used to measure sleep quality and patterns.

### MR imaging acquisition

**Fig. 1:**
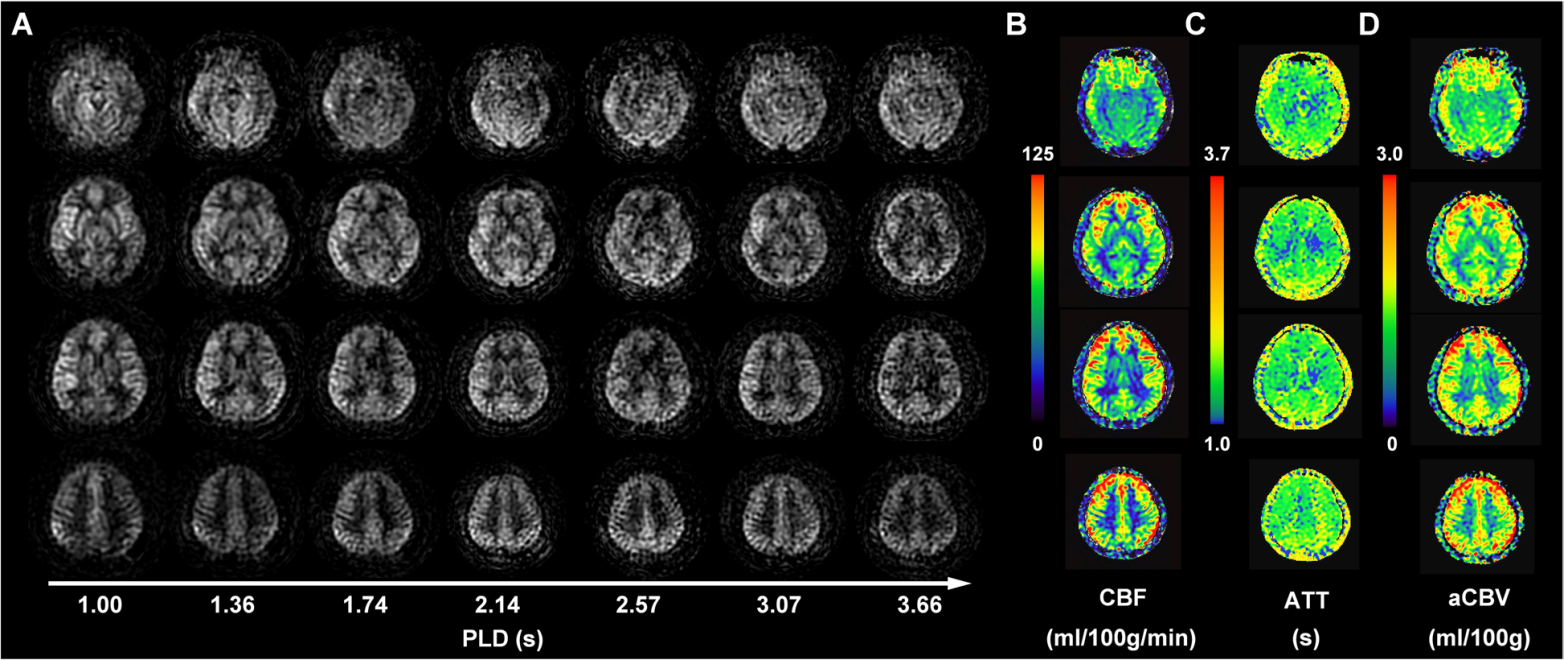
Diagram of cerebral perfusion by multi-delay ASL MR imaging. (**A**) The raw maps of ASL imaging, including different post label delays (PLDs) ranging from 1.00 to 3.66 seconds (s). (**B**) The arrival-time-corrected cerebral blood flow (CBF) colormaps. (**C**) The arterial arrival time (ATT) colormaps. (**D**) The arterial cerebral blood volume (aCBV) colormaps

MR imaging was performed on a 3 T MR scanner (Signa Premier, GE Healthcare, Waukesha, WI) using a 48-channel head coil. All participants were instructed to lie in a supine position, and formed padding was used to limit head movement. Volumetric perfusion imaging was obtained using a multi-delay pCASL sequence with spiral readout. The parameters were as follows: TR = 7138 ms; TE = 11 ms; slice thickness = 4.5 mm; NEX = 1; readout: 5 arms × 640 samples; FOV = 208 mm × 208 mm, and reconstruction matrix = 128 × 128. The total examination time for the ASL protocol was 4 minutes 44 seconds. This protocol encodes seven different post-labeling delay (PLD) times into a single acquisition. Images with PLD times of 1.00, 1.36, 1.74, 2.14, 2.57, 3.07, and 3.66 seconds and effective label durations (LD) of 0.36, 0.38, 0.40, 0.44, 0.49, 0.59, and 0.84 seconds were acquired (Fig. 1).

### Imaging analysis

The arterial transit time (ATT) maps can be estimated with signal weighted delay described by Dai et al. [30] For each pair of PLD and LD, the arrival-time-corrected CBF maps can be quantified as follows:

$$CBF=\frac{6000{e}^{\delta /{T}_{1a}}}{2\epsilon {T}_{1a}\left({e}^{-\frac{\max \left(\omega -\delta, 0\right)}{T_{1a}}}-{e}^{-\frac{\max \left(\tau +\omega -\delta, 0\right)}{T_{1a}}}\right)}\frac{M}{M0},$$

in which *δ* is the arterial transit time, *τ* is the LD, *ω* is the PLD, *T*_1*a*_ is the longitudinal relaxation time of arterial blood (1.6 s), *ϵ* is the combined efficiency of labeling and background suppression (0.63), *M* is the signal intensity of the perfusion weighted image, and M0 is the signal intensity of the reference image. The final CBF was the mean of the estimated CBF at each pair of PLD and LD. Arterial cerebral blood volume (aCBV) maps were generated by the product of ATT and CBF, which indicates the arterial blood volume from the labeling plane to the imaging voxel [31]:

$$aCBV= CBF\bullet ATT$$

The arrival-time-corrected CBF and ATT maps were registered to the standard Montreal Neurologic Institute (MNI) stereotaxic space using Statistical Parametric Mapping 12 (SPM 12) (http://www.fil.ion.ucl.ac.uk/spm/) with the aid of M0 images acquired in ASL sequence. The automated anatomical labelling atlas 3 (AAL3) [32] was used to parcellate the brain into 170 regions. The mean CBF, ATT and aCBV values of gray matter in each region were calculated. The visualization of arrival-time-corrected CBF, ATT and aCBV colormaps is presented in Fig. 1.

## Statistical analysis

The sample size was based on the available data and previous literature. A sample size of 30 cases (15 HC group and 15 CM group) would provide 80% power to reject the null hypothesis equal means when the mean difference is 5.49 (55.83–49.34) with standard deviations of 6.55 for HC group and 6.09 for CM group at a two-sided alpha of 0.05 [10]. Given an anticipated dropout rate of 20%, the total sample size required is 36 cases (18 HC group and 18 CM group). Fifteen NDPH cases were included in this study according to the previous similar studies [10, 21, 22, 33, 34]. To match the age and sex of patients with NDPH, 15 HC and 18 patients with CM were included in this study. All quantitative data were expressed as mean ± standard deviation (SD) for the normal distribution data or median with a range for the non-normal distribution data. The Kolmogorov–Smirnov test was used to test the normality of clinical data and cerebral perfusion parameters. Categorical variables were analyzed using the Chi-square test or Fisher’s exact test. For the normally distributed data, comparisons of CBF and aCBV values among the three groups (HC, NDPH, and CM) were performed by one-way analysis of variance (ANOVA), and post hoc analysis with the Bonferroni correction method [35] was used for multiple comparisons. For the non-normally distributed data, comparisons of CBF and aCBV values in brain regions among the three groups were performed by the Kruskal-Wallis H test, and all pairwise comparisons were performed using Kruskal-Wallis 1-way ANOVA (k samples). Comparisons of clinical characteristics between the CM and NDPH groups were performed by the Independent Samples T-test for the normally distributed data and the Mann-Whitney U test for the non-normally distributed data. The correlations between clinical characteristics and brain regions with significant differences were determined using Pearson’s or Spearman’s correlation analysis with age and sex as covariates, depending on whether the data were normally or non-normally distributed. Positive and negative values of correlation coefficient *r* represent positive and negative correlations. A two-sided *P* < 0.05 was considered statistically significant. All statistical analyses were performed using SPSS 26.0 software (SPSS Inc., Chicago, IL, USA).

## Results

### Patient demographics and clinical characteristics

Fifty-one participants (16 HC, 15 NDPH, and 20 CM participants) were enrolled in this study. The patient enrollment flowchart is shown in Supplementary Fig. 1. Two patients with CM were excluded due to poor images (*n* = 2). And one HC participant was excluded due to the poor images. In total, 48 participants, including 15 HC, 15 NDPH, and 18 CM participants, were included in this study. Demographics and clinical characteristic data in different groups are summarized in Table 1. All participants were right-handed. No significant differences were found in terms of age, sex, and body mass index. Compared with NDPH patients, CM patients had less frequency of bilateral headache (*P* = 0.007), more severe headache intensity (*P* = 0.003), and a higher frequency of light sensitivity (*P* = 0.047) and vomiting (*P* = 0.012). There were no significant differences in other clinical characteristics among different groups.

**Table 1:** Demographic characteristics and clinical data of participants in the three groups

	HC (***n*** = 15)	CM (***n*** = 18)	NDPH (***n*** = 15)	***P*** value
Age (years)	40.93 ± 9.11	41.61 ± 12.66	44.93 ± 17.15	0.677
Female, *n* (%)	8 (53.3)	11 (61.1)	7 (46.7)	0.707
BMI (kg/m^2^)	22.84 ± 2.92	23.44 ± 3.62	23.79 ± 3.63	0.746
Right-handers, *n* (%)	15 (100.0)	18 (100.0)	15 (100.0)	1.000
Headache laterality, *n* (%)				
Unilateral	NA	7 (38.9)	3 (20.0)	0.426
Bilateral	NA	6 (33.3)	12 (80.0)	0.007**
Shift	NA	5 (27.8)	0 (0.0)	**NA**
Location of headache, *n* (%)				
Frontal region	NA	10 (55.6)	5 (33.3)	0.202
Temporal region	NA	14 (77.8)	11 (73.3)	> 0.999
Parietal region	NA	12 (66.7)	9 (60.0)	0.692
Occipital region	NA	10 (55.6)	6 (40.0)	0.373
Periorbital region	NA	5 (27.8)	0 (0.0)	**NA**
Disease duration (years)	NA	20.44 ± 8.97	14.52 ± 14.23	0.155
Headache frequency, days/month	NA	30.00 (15.75–30.00)	NA	**NA**
Headache intensity^a^	NA	7.44 ± 1.38	5.67 ± 1.84	0.003**
Light sensitivity, *n* (%)	NA	16 (88.9)	8 (53.3)	0.047*
Noise sensitivity, *n* (%)	NA	15 (83.3)	9 (60.0)	0.239
Vomiting, *n* (%)	NA	10 (55.6)	2 (13.3)	0.012*
HIT-6 score (36–78)	NA	64.53 ± 9.64	64.36 ± 9.93	0.962
PHQ-9 score (0–27)	NA	7.50 (3.50–16.00)	10.00 (7.00–17.00)	0.247
GAD-7 score (0–21)	NA	4.50 (2.00–9.75)	6.00 (5.00–10.00)	0.140
PSQI score (0–21)	NA	9.86 ± 4.37	11.50 ± 3.55	0.284

*HC* healthy control, *NDPH* new daily persistent headache, *CM* chronic migraine, *NA* not applicable. *BMI* body mass index, *HIT-6* Headache Impact Test-6, *PHQ-9* Patient Health Questionnaire-9, *GAD-7* Generalized Anxiety Disorder-7, *PSQI* Pittsburgh Sleep Quality Index, ^*a*^ Headache intensity on a 0–10 numerical rating scale. * *P*<0.05, ** *P*<0.01

### Comparison of regional CBF values among HC, NDPH and CM groups

**Fig. 2:**
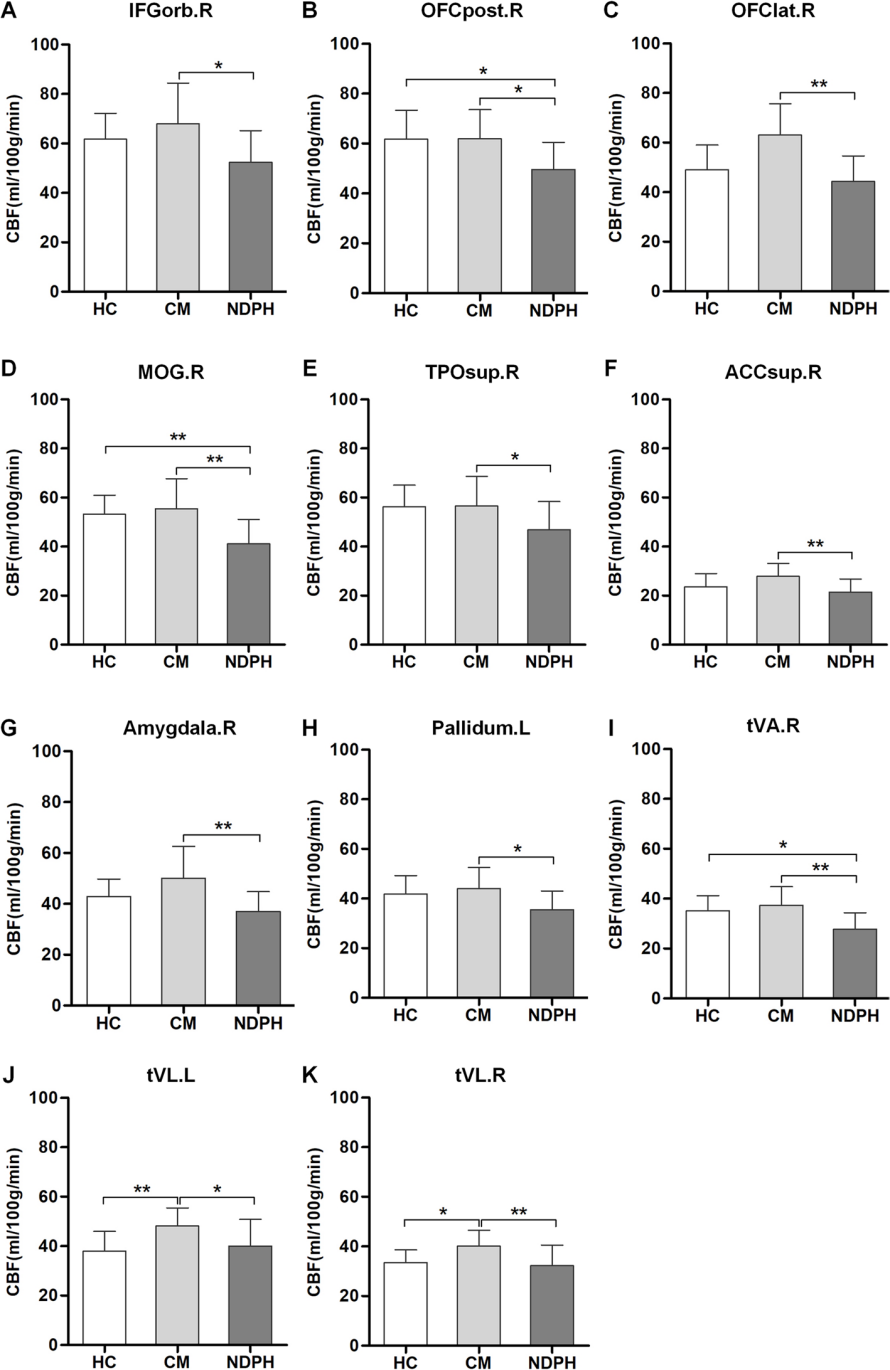
Brain regions with significant differences in CBF between NDPH and HC groups, NDPH and CM groups, as well as CM and HC groups (**A-K**). *HC* healthy control, *NDPH* new daily persistent headache, *CM* chronic migraine. *IFGorb.R* right inferior frontal gyrus pars orbitalis, *OFCpost.R* right posterior orbital gyrus, *OFClat.R* right lateral orbital gyrus, *ACCsup.R* right anterior cingulate cortex, supracallosal gyrus. *TPOsup.R* right superior temporal gyrus, temporal pole, *MOG.R* right middle occipital gyrus, *Amygdala.R* right amygdala, *Pallidum.L* left pallidum, *tVA.R* right thalamus, ventral anterior nucleus, *tVL.L* left thalamus, ventral lateral nucleus, *tVL.R* right thalamus, ventral lateral nucleus. * *P*<0.05, ** *P*<0.01

As shown in Table 2, there were significant differences in regional CBF values among HC, NDPH and CM participants (all *P* < 0.05). This study found six brain regions with significant differences in the cerebral cortex and five in the deep nuclei. Compared with HC participants, the CBF was decreased in the right posterior orbital gyrus (OFCpost.R) (*P* = 0.023), right middle occipital gyrus (MOG.R) (*P* = 0.002), and ventral anterior nucleus of the right thalamus (tVA.R) (*P* = 0.014) in the patients with NDPH. In the contrast, for patients with CM, the CBF was increased in the ventral lateral nucleus of the left thalamus (tVL.L) (*P* = 0.006) and right thalamus (tVL.R) (*P* = 0.023) compared with HC participants. Compared with CM, the regional CBF was observed to be significantly decreased in NDPH (all *P* < 0.05) (Fig. 2).

**Table 2:** Brain regions with significant differences in CBF among different groups

Locations	Brain	CBF (ml/100 g/min)			
	Regions	HC (***n*** = 15)	CM (***n*** = 18)	NDPH (***n*** = 15)	***P*** value
Cortex	IFGorb.R	58.82 (55.96–71.27)	67.44 (57.93–79.50)	47.88 (43.46–58.29)	0.008**
	OFCpost.R	58.77 (53.01–68.60)	60.67 (57.14–72.10)	46.42 (43.44–56.43)	0.004**
	OFClat.R	49.03 ± 10.00	60.25 ± 17.02	44.26 ± 10.39	0.003**
	ACCsup.R	25.13 (22.59–27.60)	27.22 (24.03–31.80)	21.64 (16.89–27.11)	0.012*
	TPOsup.R	56.32 ± 8.68	56.56 ± 12.11	46.86 ± 11.54	0.026*
	MOG.R	53.10 (46.97–60.14)	54.10 (46.42–68.23)	39.34 (34.91–43.86)	< 0.001***
Nucleus	Amygdala.R	41.31 (39.28–48.45)	46.47 (43.60–57.97)	35.28 (30.33–42.69)	0.002**
	Pallidum.L	41.76 ± 7.52	44.07 ± 8.47	35.54 ± 7.54	0.011*
	tVA.R	35.13 ± 6.04	37.31 ± 7.47	27.76 ± 6.63	0.001**
	tVL.L	38.06 ± 7.89	48.10 ± 7.39	40.08 ± 10.78	0.004**
	tVL.R	33.58 ± 5.04	40.12 ± 6.44	32.17 ± 8.35	0.003**

*HC* healthy control, *NDPH* new daily persistent headache, *CM* chronic migraine. *IFGorb.R* right inferior frontal gyrus pars orbitalis, *OFCpost.R* right posterior orbital gyrus, *OFClat.R* right lateral orbital gyrus, *ACCsup.R* right anterior cingulate cortex, supracallosal gyrus, *TPOsup.R* right superior temporal gyrus, temporal pole, *MOG.R* right middle occipital gyrus, *Amygdala.R* right amygdala, *Pallidum.L* left pallidum, *tVA.R* right thalamus, ventral anterior nucleus, *tVL.L* left thalamus, ventral lateral nucleus, *tVL.R* right thalamus, ventral lateral nucleus. * *P*<0.05, ** *P*<0.01,*** *P*<0.001

### Comparison of regional aCBV values among HC, NDPH and CM groups

**Fig. 3:**
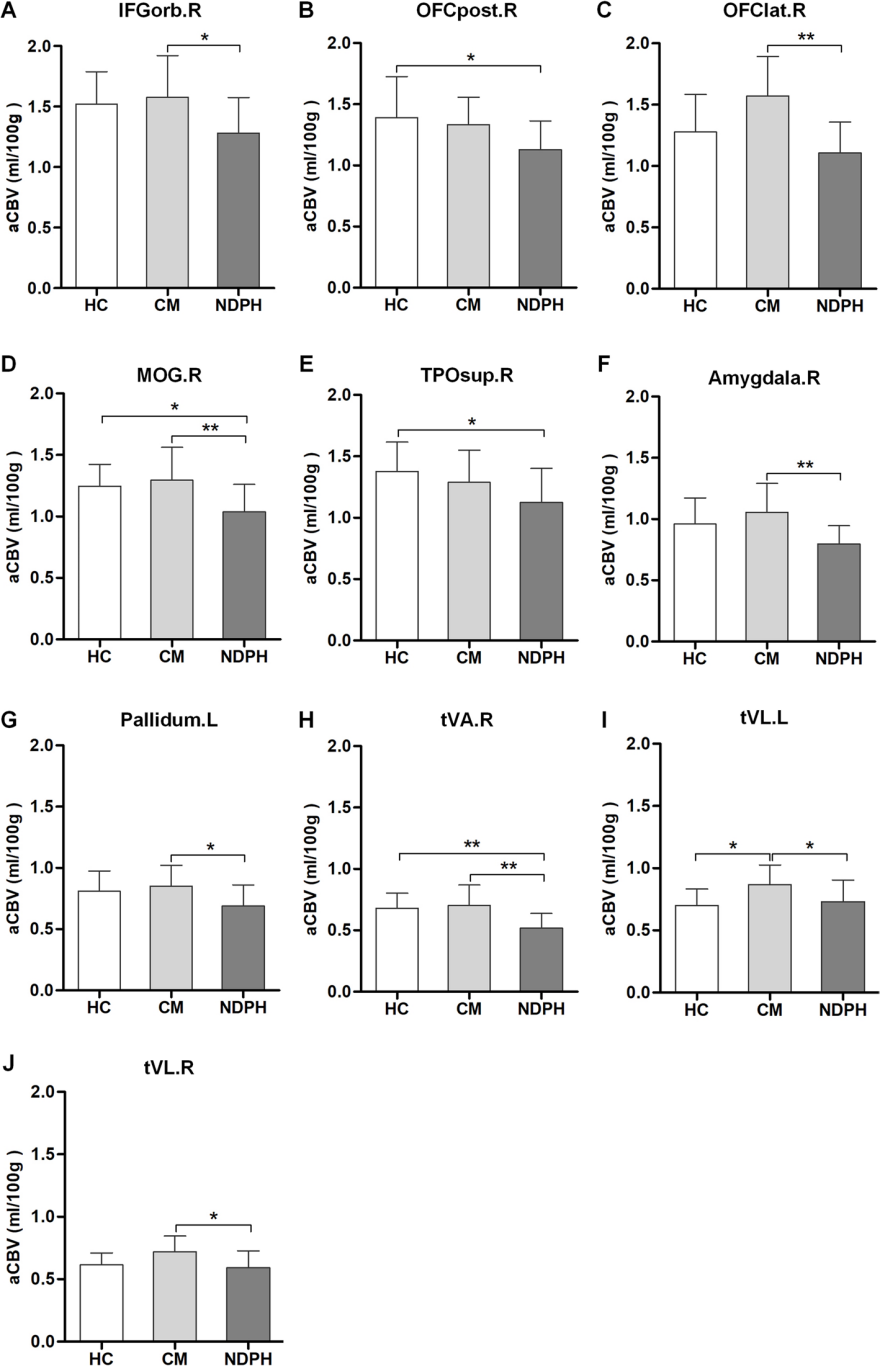
Brain regions with significant differences in the aCBV between NDPH and HC groups, NDPH and CM groups, as well as CM and HC groups (**A-J**). *HC* healthy control, *NDPH* new daily persistent headache, *CM* chronic migraine. *IFGorb.R* right inferior frontal gyrus pars orbitalis, *OFCpost.R* right posterior orbital gyrus, *OFClat.R* right lateral orbital gyrus, *TPOsup.R* right superior temporal gyrus, temporal pole, *MOG.R* right middle occipital gyrus, *Amygdala.R* right amygdala, *Pallidum.L* left pallidum, *tVA.R* right thalamus, ventral anterior nucleus, *tVL.L* left thalamus, ventral lateral nucleus, *tVL.R* right thalamus, ventral lateral nucleus. * *P*<0.05, ** *P*<0.01

**Fig. 4:**
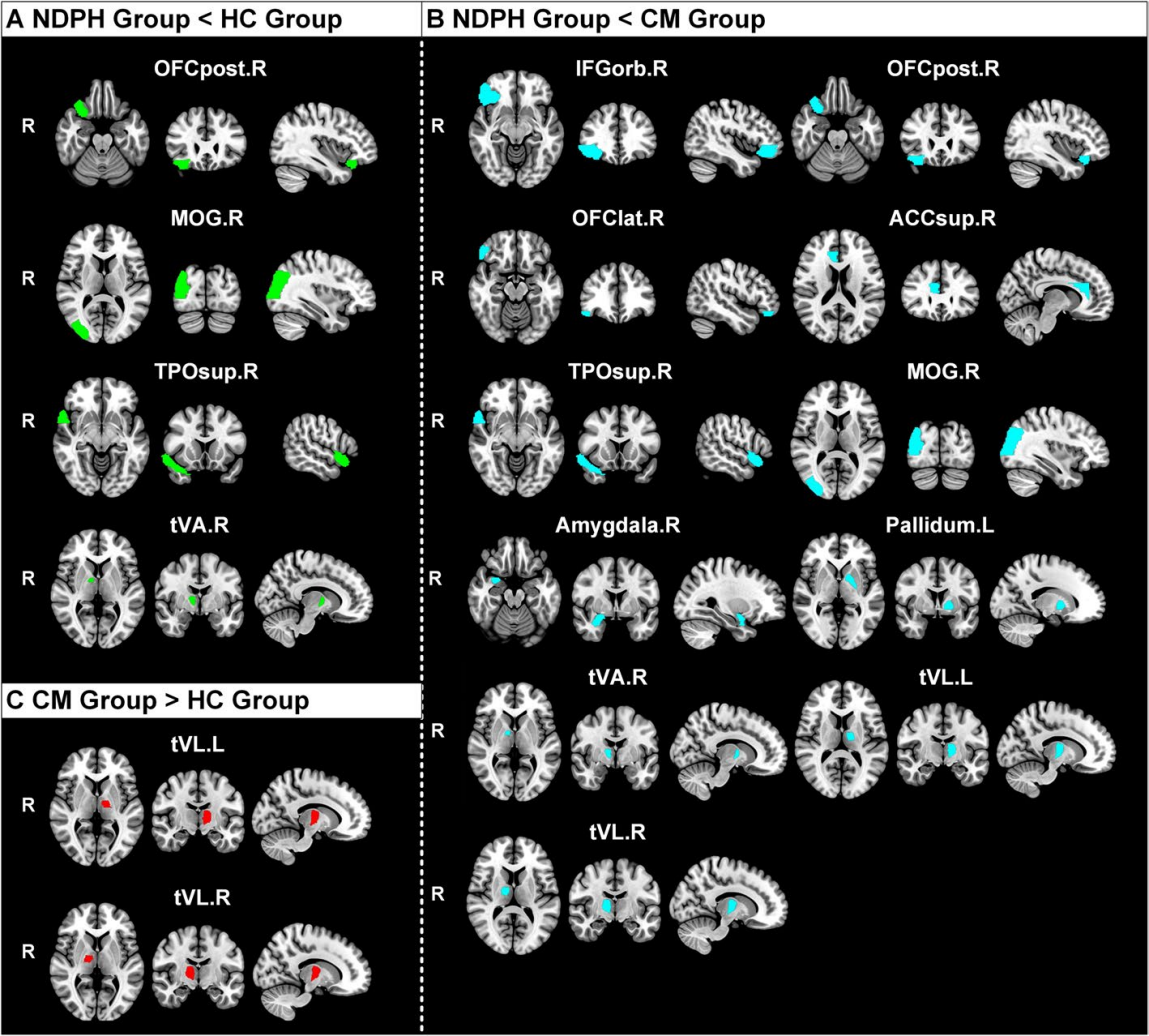
The visualization of brain regions with significant differences in cerebral perfusion between NDPH and HC groups, CM and HC groups, as well as NDPH and CM groups. The brain regions with decreased cerebral perfusion in NDPH groups were presented compared with HC (**A**) and CM (**B**) groups, respectively. The brain regions with increased cerebral perfusion in CM groups were presented compared with HC groups (**C**). *HC* healthy control, *NDPH* new daily persistent headache, *CM* chronic migraine. *IFGorb.R* right inferior frontal gyrus pars orbitalis, *OFCpost.R* right posterior orbital gyrus, *OFClat.R* right lateral orbital gyrus, *ACCsup.R* right anterior cingulate cortex, supracallosal gyrus, *TPOsup.R* right superior temporal gyrus, temporal pole, MOG.R right middle occipital gyrus, *Amygdala.R* right amygdala, *Pallidum.L* left pallidum, *tVA.R* right thalamus, ventral anterior nucleus, *tVL.L* left thalamus, ventral lateral nucleus, *tVL.R* right thalamus, ventral lateral nucleus

Significant differences in regional aCBV values among HC, NDPH, and CM participants are presented in Table 3. Compared with HC participants, the aCBV was decreased in the right posterior orbital gyrus (OFCpost.R) (*P* = 0.030), right middle occipital gyrus (MOG.R) (*P* = 0.048), temporal pole of right superior temporal gyrus (TPOsup.R) (*P* = 0.034), and ventral anterior nucleus of the right thalamus (tVA.R) (*P* = 0.009) in patients with NDPH. On the contrary, for patients with CM, the aCBV was raised in the ventral lateral nucleus of the left thalamus (tVL.L) (*P* = 0.010) compared with HC participants. Compared with CM, the regional aCBV was significantly decreased in NDPH (all *P* < 0.05), except in the OFCpost.R, TPOsup.R, and right anterior cingulate cortex, supracallosal gyrus (ACCsup.R) (Fig. 3). As shown in Fig. 4, the visualization of brain regions with significant differences in cerebral perfusion was performed between the NDPH and HC groups, NDPH and CM groups, as well as the CM and HC groups.

**Table 3:** Brain regions with significant differences in aCBV among different groups

Locations	Regions	aCBV (ml/100 g)			
		HC (***n*** = 15)	CM (***n*** = 18)	NDPH (***n*** = 15)	***P*** value
Cortex	IFGorb.R	1.52 ± 0.27	1.58 ± 0.34	1.28 ± 0.29	0.022*
	OFCpost.R	1.39 ± 0.34	1.33 ± 0.22	1.13 ± 0.24	0.024*
	OFClat.R	1.28 ± 0.31	1.50 ± 0.43	1.11 ± 0.25	0.009**
	ACCsup.R	0.48 ± 0.11	0.56 ± 0.13	0.45 ± 0.13	0.043*
	TPOsup.R	1.38 ± 0.24	1.28 ± 0.26	1.12 ± 0.28	0.035*
	MOG.R	1.25 ± 0.18	1.29 ± 0.27	1.04 ± 0.22	0.006**
Nucleus	Amygdala.R	0.88 (0.85–1.12)	1.01 (0.86–1.21)	0.76 (0.69–0.87)	0.002**
	Pallidum.L	0.81 ± 0.17	0.85 ± 0.17	0.69 ± 0.17	0.028*
	tVA.R	0.68 ± 0.14	0.70 ± 0.17	0.52 ± 0.12	0.001**
	tVL.L	0.70 ± 0.13	0.87 ± 0.16	0.73 ± 0.17	0.007**
	tVL.R	0.61 ± 0.10	0.71 ± 0.13	0.59 ± 0.14	0.009**

*HC* healthy control, *NDPH* new daily persistent headache, *CM* chronic migraine. *IFGorb.R* right inferior frontal gyrus pars orbitalis, *OFCpost.R* right posterior orbital gyrus, *OFClat.R* right lateral orbital gyrus, *ACCsup.R* right anterior cingulate cortex, supracallosal gyrus, *TPOsup.R* right superior temporal gyrus, temporal pole, *MOG.R* right middle occipital gyrus, *Amygdala.R* right amygdala, *Pallidum.L* left pallidum, *tVA.R* right thalamus, ventral anterior nucleus, *tVL.L* left thalamus, ventral lateral nucleus, *tVL.R* right thalamus, ventral lateral nucleus. * *P*<0.05, ** *P*<0.01

### Correlation analysis between cerebral perfusion parameters in brain regions with significant differences and clinical variables

**Fig. 5:**
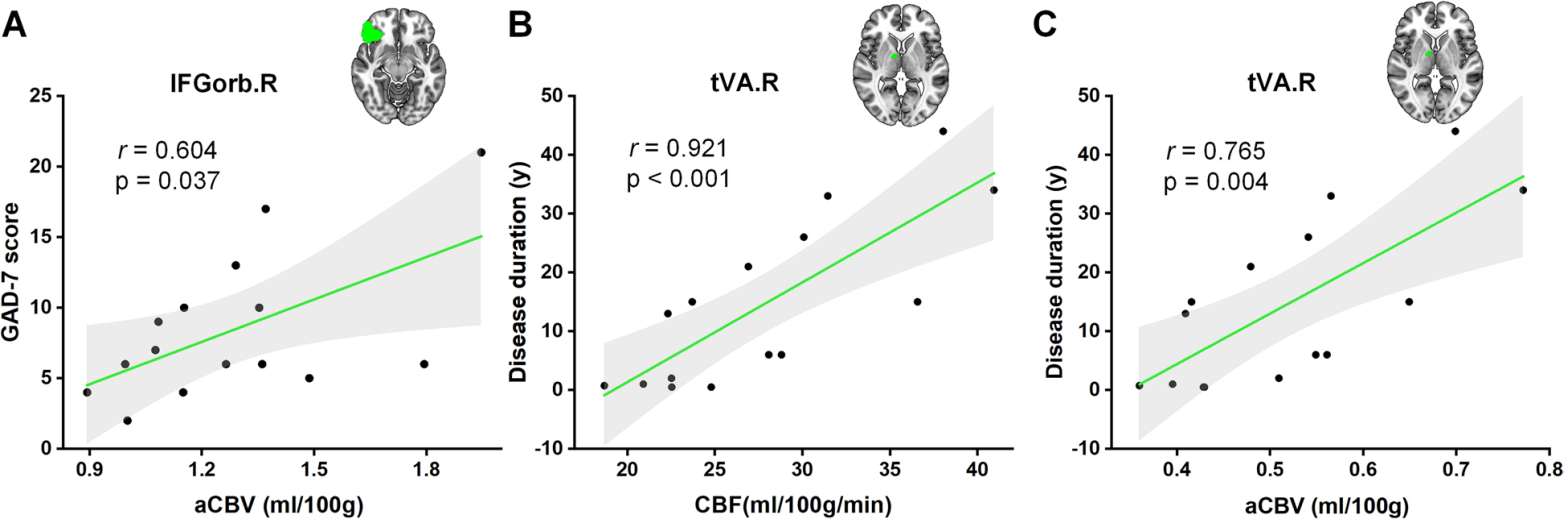
Correlation analysis between cerebral perfusion parameters and clinical variables in brain regions with significant differences of NDPH. The aCBV of the right inferior frontal gyrus pars orbitalis (IFGorb.R) was positively correlated with the GAD-7 score (**A**). The CBF of the right thalamus, ventral anterior nucleus (tVA.R) was positively correlated with disease duration (**B**), as well as the aCBV of tVA.R (**C**). *NDPH* new daily persistent headache, *aCBV* arterial cerebral blood volume, *CBF* cerebral blood flow, *GAD-7* Generalized Anxiety Disorder-7

For patients with NDPH, the correlation analysis showed that the increased aCBV in the region of IFGorb.R was positively correlated with the GAD-7 score (*r* = 0.604, *P* = 0.037, *n* = 15) after the age and sex adjustments. Meanwhile, the increased CBF (*r* = 0.921, *P* < 0.001, n = 15) and aCBV (*r* = 0.765, *P* = 0.004, *n* = 15) in the tVA.R region were positively correlated with disease duration (Fig. 5). No significant correlation was found between cerebral perfusion parameters and other clinical characteristics in the brain regions with significant differences (all *P* > 0.05). For patients with CM, there were no significant correlations between cerebral perfusion parameters and clinical characteristics in the brain regions with significant differences (all *P* > 0.05).

## Discussion

In the present study, we found that, compared with HCs, patients with NDPH showed a decrease in CBF or aCBV values in multiple cortical regions in the right hemisphere, while patients with CM showed an increase in CBF and aCBV values in the bilateral thalamus. These intriguing findings may reveal the different cerebral hemodynamic variations between these two types of primary chronic headache. So far, the underlying neurovascular mechanism of NDPH is still unknown. There has been no report on the cerebral hemodynamic features of patients with NDPH. Only a few structural imaging studies on NDPH was reported. A recent study showed that patients with NDPH had no structural brain changes [36]. Another study of adolescent found that patients with NDPH reduced cortical thickness in the bilateral superior temporal gyrus, left superior, and middle frontal gyrus areas compared with controls [34]. There is still some controversy regarding the alteration of NDPH structure. One previous study of chronic tension-type headache (CTTH) showed a significant gray matter volume decrease in pain processing regions [37]. Meanwhile, Schmidt-Wilcke et al. [37] revealed the brain structural levels are different between patients with CTTH and migraine. Similarly, from the perspective of cerebral perfusion in NDPH and CM, our study found that the different regional perfusion between the two types of primary chronic headache.

Previous studies have reported the cerebral hyperperfusion pattern of episodic migraine (EM) in several brain regions [12, 21, 22]. Another study on tinnitus patients with migraine showed reduced CBF in the temporal and prefrontal cortex [38]. One recent study of CM using 3D pCASL imaging detected hypoperfusion of the left nucleus accumbens [10]. However, cerebral hemodynamic investigation was still rarely reported in CM. The current study showed hyperperfusion in the bilateral thalamus for patients with CM. Thalamus is considered the relay center for ascending nociceptive information [39]. According to functional imaging studies, the increased thalamus neuronal activation during migraine attacks and the overall abnormal functional connectivity in the thalamocortical limb of the trigeminovascular pathway suggested dysfunctional pain processing in migraine [40, 41]. For patients with CM, during the process of migraine chronification, recurring attacks of migraine may lead to increase neuronal activation in the thalamus, which may be one of the reasons for cerebral hyperperfusion in the thalamus. One magnetic resonance spectroscopy (MRS) study in migraineurs revealed significantly increased glutamate/glutamine (GLX) levels in both the primary occipital cortex and right thalamus [42]. The changes in brain tissue metabolism levels might reflect cerebral perfusion variance to some extent.

Altered regional cerebral perfusion may reflect differences in neuronal metabolism or activity. Tumor necrosis factor alpha (TNF-α) is a proinflammatory cytokine involved in central nervous system (CNS) inflammation, immune activity, and pain initiation. Rozen et al. [43] found that 95% of NDPH patients had elevated TNF-α levels in cerebrospinal fluid (CSF) and suggested a role of TNF-α in the pathogenesis of NDPH. Calcitonin gene-related peptide (CGRP) is a known factor in the migraine pathogenesis cascade [44]. Previous evidence showed that TNF-α would induce CGRP production [45]. It has been speculated that intracranial TNF-α receptors are located in trigeminal ganglion neurons, and the release of TNF-α leads to an increase in CGRP levels. CGRP has been confirmed to cause the vasodilation of meninges and intracranial arteries and contribute to neurogenic inflammation by triggering the release of neuron sensitizing agents from mast cells [44, 46, 47]. As a pain factor, it participates in the transmission of intracranial blood vessels to trigeminal sensory nerve signals, leading to headache attacks. Regarding NDPH, reduced cortical cerebral perfusion may be associated with compensatory vasoconstriction due to persistent headache. A previous study reported two cases of NDPH-like headaches after acute bouts of reversible cerebral vasoconstriction syndrome (RCVS), which suggested that vasoconstriction in RCVS may be regarded as a trigger for NDPH-like headache [48]. In addition, the persistence of headache attacks might accelerate the decreased cerebral perfusion in NDPH. This is an initial observation that must be substantiated by future studies.

In our study, we found that the hypoperfusion brain regions in NDPH were lateralized to the right hemisphere, which was similar to the previous studies showing that abnormal cerebral perfusion regions in episodic migraine were lateralized to the right cortex [12, 22]. A previous multimodal MRI study of neurovascular coupling (NVC) dysfunction in CM showed that NVC biomarkers were significantly higher in right superior occipital gyrus, right superior parietal gyrus, and precuneus, which was considered the compensatory response [49]. One survey of 188 consecutive chronic headache patients reported that about 50% of headache cases were lateralized with an overall right-sided predominance (59%) [50]. One recent study reported that 62.8% of unilateral pain episodes occurred on the right side for right-handed migraine patients. This study suggested that the manual dominance of participants with migraine may strongly influence pain lateralization [51]. All patients with NDPH in this study were right-handed, which may be one of the causes of hypoperfusion in the right hemisphere. However, more frequency of bilateral headaches was presented in patients with NDPH in this study. Therefore, the lateralization of hypoperfusion brain regions in patients with NDPH needs to be investigated and verified in future studies.

In the present study, NDPH patients exhibited a decreased cerebral perfusion in the prefrontal cortex (PFC), including IFGorb.R and OFCpost.R, which are the critical areas of reward, decision making, and emotion regulation [52, 53]. A previous study revealed that NDPH is linked to anxiety and panic disorders [54]. Patients with NDPH showed defects in emotional expression after impaired PFC function. Our study found that the increased aCBV of IFGorb.R was positively correlated with the GAD-7 score in NDPH, which were similar to a previous study [55]. In addition, the increased CBF and aCBV of tVA.R were positively correlated with disease duration. A possible explanation is that with the prolongation of the disease duration, NDPH manifested as a decreased vasoconstriction response, and the chronification process of NDPH may be a possible reason for the increased regional cerebral perfusion. However, the pathogenesis of NDPH and the relationship between cerebral perfusion and clinical features have not been clearly reported. Our findings need to be confirmed by future studies.

The present study used multi-delay pCASL MR imaging, a novel non-enhancement perfusion sequence, to detect changes in cerebral perfusion of NDPH and CM. As far as we know, it is the first study to utilize multi-delay pCASL MRI for assessing the cerebral perfusion of NDPH. Multi-delay pCASL, in which images with several post-label delay times are acquired, has several potential advantages over existing single delay ASL scans, including improved accuracy of CBF quantification, imaging of multiple hemodynamic parameters (ATT, and CBF), and better visualization of collateral flow through dynamic image series [31]. Previous ASL studies generally employed a single post-labeling delay (PLD) time typically between 1.5 and 2 s for the estimation of CBF [10, 12]. However, prolonged ATT may result in underestimation of CBF in brain tissue. In this study, we encoded seven different PLD times with the application of seven effective label durations. Many studies on cerebrovascular disease have utilized multi-delay ASL approaches to correct CBF values for arrival time [56–58]. It was demonstrated that the multi-delay ASL technique holds an advantage for clinical applications, particularly for patients with arterial transit time abnormalities.

There were several limitations to our study. First, the sample size was relatively small, which might lead to the resulting bias in this study. However, NDPH cases are not common in clinical practice. Also, patients with NDPH had a wide range of headache duration, which caused the heterogeneity of patients. But there was no significant difference in the headache duration between the NDPH and CM groups in this study. Nonetheless, the small sample size and heterogeneity of patients may affect the generalizability of these results. This was a preliminary study to investigate the cerebral perfusion variance of NDPH and CM. We will increase the sample size to verify the accuracy and generalization of the results in future studies. In addition, it was a cross-sectional study, with only CM without aura included. Further studies will follow with a focus on the dynamic perfusion changes during different phases of a CM attack and post-attack. Moreover, the spatial resolution of ASL imaging used in the current study was 1.6 mm × 1.6 mm × 4.0 mm, which made it impossible to investigate the changes in the substructure of small nuclei. However, improving the spatial resolution will significantly decrease the SNR and prolong the acquisition time. Further technical development of ASL techniques is necessary to improve the accuracy of perfusion parameter quantification.

## Conclusion

The multi-delay pCASL technique can detect cerebral perfusion variation in patients with NDPH and CM. The cerebral perfusion changes may suggest different variations between NDPH and CM, which might provide hemodynamic evidence of these two types of primary headaches.

## Supplementary Information

**Additional file 1: Supplementary Fig. 1.** The patient enrollment flowchart.

## Data Availability

Data can be made available upon request.

## Abbreviations

HC: Healthy control
NDPH: New daily persistent headache
CM: Chronic migraine
EM: Episodic migraine
CTTH: Chronic tension-type headache
pCASL: Pseudo-continuous arterial labeling
PLD: Post-label delay
CBF: Cerebral blood flow
ATT: Arterial transit time
aCBV: Arterial cerebral blood volume
IFGorb.R: Right inferior frontal gyrus pars orbitalis
OFCpost.R: Right posterior orbital gyrus
OFClat.R: Right lateral orbital gyrus
ACCsup.R: Right anterior cingulate cortex, supracallosal gyrus
TPOsup.R: Right superior temporal gyrus, temporal pole
MOG.R: Right middle occipital gyrus
Amygdala.R: Right amygdala
Pallidum.L: Left pallidum
tVA.R: Right thalamus, ventral anterior nucleus
tVL.L: Left thalamus, ventral lateral nucleus
tVL.R: Right thalamus, ventral lateral nucleus
HIT-6: Headache Impact Test-6
PHQ-9: Patient Health Questionnaire-9
GAD-7: Generalized Anxiety Disorder-7
PSQI: Pittsburgh Sleep Quality Index
TNF-α: Tumor necrosis factor alpha
CGRP: Calcitonin gene-related peptide
RCVS: Cerebral vasoconstriction syndrome
CSF: Cerebrospinal fluid
SNR: Signal-to-noise ratio
PET: Positron emission tomography
DSC: Dynamic susceptibility contrast
ICHD-3: International Classification of Headache Diseases, 3rd edition
MNI: Montreal Neurologic Institute
SPM 12: Statistical Parametric Mapping 12
AAL3: Automated anatomical labelling atlas 3
MRI: Magnetic resonance imaging
ANOVA: One-way analysis of variance
MRS: Magnetic resonance spectroscopy
GLX: Glutamate/glutamine
CNS: Central nervous system
NVC: Neurovascular coupling
PFC: Prefrontal cortex
