# Supplementary figures and images for: Cerebral perfusion variance in new daily persistent headache and chronic migraine: an arterial spin-labeled MR imaging study
Source: J Headache Pain. 2022 Dec 8;23(1):156. doi: 10.1186/s10194-022-01532-7 (PMC9733035; doi:10.1186/s10194-022-01532-7)

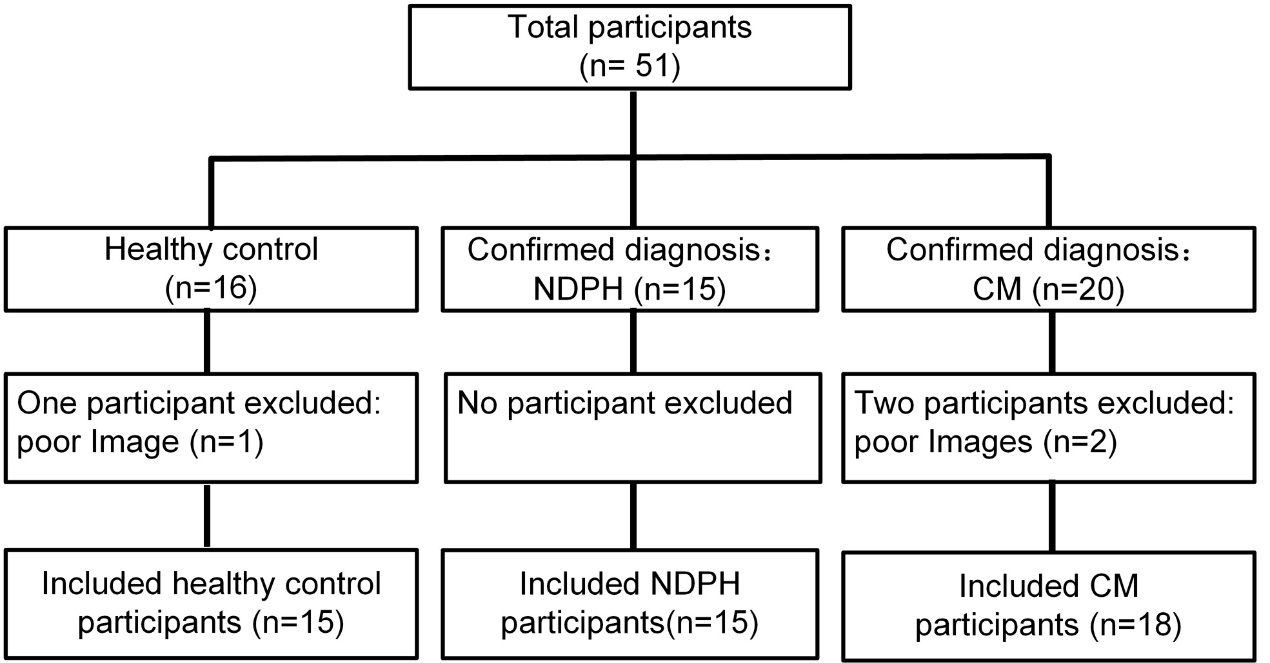


**Supplementary Figure 1**. The patient enrollment flowchart.

Supplement: Supplementary file 1 — Additional file 1: Supplementary Fig. 1. The patient enrollment flowchart. [file 10194_2022_1532_MOESM1_ESM.docx]
